# Increased Blood-Brain Barrier Permeability and Cognitive Impairment in Patients With ESKD

**DOI:** 10.1016/j.ekir.2024.07.021

**Published:** 2024-07-20

**Authors:** Mickaël Bobot, Eric Guedj, Noémie Resseguier, Julien Faraut, Philippe Garrigue, Vincent Nail, Guillaume Hache, Sandra Gonzalez, Nathalie McKay, Romain Vial, Dammar Bouchouareb, Guillaume Lano, Noémie Jourde-Chiche, Ariane Duval-Sabatier, Fabrice Guilaume, Benjamin Guillet, Stéphane Burtey

**Affiliations:** 1 Centre de Néphrologie et Transplantation Rénale, Hôpital de la Conception, Marseille, France; 2 Aix Marseille Université, INSERM 1263, INRAE 1260, C2VN, Marseille, France; 3 CERIMED, Aix-Marseille Université, Marseille, France; 4 APHM, CNRS, Centrale Marseille, Institut Fresnel, Timone Hospital, CERIMED, Nuclear Medicine Department, Aix Marseille Université, Marseille, France; 5 CEReSS/UR 3279 - Health Services and Quality of Life Research, Aix Marseille University, Marseille, France; 6 Methodological Support Unit for Clinical and Epidemiological Research, University Hospital of Marseille, Marseille, France; 7 Radiopharmacie, Marseille, France; 8 Pharmacie, Hôpital de la Timone, Marseille, France; 9 Association des Dialysés Provence et Corse, Marseille, France; 10 Centre de Recherche en Psychologie et Neuroscience, CNRS, UMR7077, Aix-Marseille Université, Marseille, France

**Keywords:** blood-brain barrier, chronic kidney disease, cognitive impairment, indoxyl sulphate, kidney-brain axis, uremic toxins

## Abstract

**Introduction:**

Chronic kidney disease (CKD) is associated with an increased risk of cognitive impairment. This cognitive impairment is associated with an increased permeability of blood-brain barrier (BBB) in rodents with CKD, linked to activation of aryl hydrocarbon receptor (AhR) by indoxyl sulphate (IS). The objective of the BREIN study was to confirm the increased BBB permeability in humans with CKD.

**Method:**

The BREIN comparative study (NCT04328415) prospectively included patients with end-stage kidney disease (ESKD) and controls healthy volunteers matched in age, sex, and level of education to a patient. In all participants, BBB permeability was quantified by brain ^99m^Tc-DTPA SPECT/CT as a percentage of injected activity (% IA). A battery of neurocognitive tests was performed, and serum uremic toxins accumulation and AhR activation were assessed.

**Results:**

Fifteen patients with ESKD and 14 healthy volunteers were analyzed. Patients with ESKD had higher BBB permeability compared to controls: 0.29 ± 0.07 versus 0.14 ± 0.06 %IA, *P* = 0.002. Patients with ESKD displayed lower Montreal Cognitive Assessment test (MoCA) score: 22.0 ± 5.0 versus 27.3 ± 2.8, *P* = 0.008; impaired short-term memory (doors test): 12.5 ± 3.4 versus 16.5 ± 3.4, *P* = 0.005; higher Beck depression score 8.1 ± 9.1 versus 2.7 ± 3.4, *P* = 0.046; and slightly more daily cognitive complaints: 42.5 ± 29.3 versus 29.8 ± 14.0 *P* = 0.060. Patients with ESKD displayed higher IS levels (86.1 ± 48.4 vs. 3.2 ± 1.7 μmol/l, *P* = 0.001) and AhR activating potential (37.7 ± 17.8% vs. 24.7 ± 10.4%, *P* = 0.027). BBB permeability was inversely correlated with MoCA score (*r* = −0.60, 95% confidence interval [−0.772 to −0.339], *P* = 0.001) in the overall population.

**Conclusion:**

Patients with ESKD display an increased BBB permeability compared to matched healthy volunteers. Association with uremic toxins and cognitive impairment needs to be assessed in larger cohorts of patients.

## Graphical abstract

**Figure:**
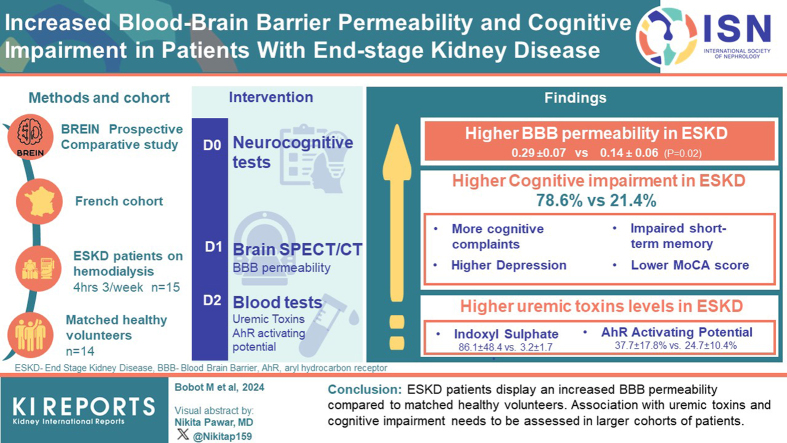

CKD is associated with an increased risk of cognitive impairment.^1^, ^2^, ^3^, ^4^ The cognitive impairment in patients with CKD appears earlier than in the general population and evolves in parallel to the decline of glomerular filtration rate.^5^^,^^6^ It could concern 30% to 80% of patients with ESKD.^1^ The clinical presentation is similar to a vascular dementia, but specific pathophysiological mechanisms are poorly understood. Along with highly prevalent traditional risk factors such as hypertension and diabetes, uremic toxin accumulation is suspected to play an important and specific role in cognitive impairment.^7^^,^^8^ During CKD, the accumulation of tryptophan-derived uremic toxins such as IS, exerts an endothelial toxicity^9^^,^^10^ leading to increased cardiovascular events.^9^^,^^11^ Several preclinical studies found that these toxins can also exert a direct neurotoxicity *in vitro*,^12^ and administration of IS was associated with altered cognitive performance in a rat model of CKD.^13^^,^^14^

The BBB is a physiological highly selective barrier separating the brain parenchyma from the blood, and guarantying its protection from circulating pathogens and neurotoxic compounds.^15^ BBB disruption appears as a major mechanism involved in neurodegenerative diseases such as Alzheimer’s disease or Parkinson’s disease^16^ but also in hypertension^17^ and diabetes,^18^ and is linked to neurocognitive impairment.^19^ Molecular isotopic imaging using diethylene-triamine-penta-acetic acid (DTPA) radiolabelled with Technetium-99m (^99m^Tc), allows a quantification of the BBB disruption in both preclinical research and clinical settings.^20^ DTPA is a small aminopolycarboxylic acid which does not cross the BBB under physiological conditions, presence of DTPA in the brain parenchyma reflects BBB disruption.

In preclinical research, BBB permeability also appears as a crucial mechanism of brain dysfunction in CKD. In several models of CKD in rats and mice, we highlighted that CKD was associated with cognitive impairment and increased BBB permeability. The BBB disruption, quantified using (^99m^Tc)-DTPA single-photon emission computed tomography coupled with computed tomography scanner (SPECT/CT) imaging, was both correlated with cognitive impairment and with IS levels. Inactivation of AhR, receptor of the IS, protected the CKD mice from the IS-induced BBB permeability.^13^

The objective of the BREIN (BBB Evaluation in Nephrology) study was to confirm an increased BBB permeability in humans with ESKD.

## Methods

This prospective comparative study was designed to include 30 participants: 15 patients with ESKD, and 15 healthy volunteers. Adult patients with ESKD undergoing chronic hemodialysis at least 4 hours 3 times a week were included. Volunteers were adults matched in age (± 5 years), sex, and educational level to a patient.

All procedures were in accordance with the ethical standards of the responsible committee on human experimentation and with the Helsinki Declaration of 1975 revised in 2000 and were previously approved by an independent research committee (CPP Ile de France 10, ref. 20-00885082929) and registered in ClinicalTrials (NCT04328415). All patients received oral and written information and provided their informed written consent to participate.

Exclusion criteria were pregnancy, incapacity of providing informed consent, history of neurodegenerative disease, stroke, no equilibrated coronary disease, ongoing infection or neoplasia, no equilibrated hypertension (systolic blood pressure > 160 mm Hg before inclusion), ongoing medication with neurocognitive consequences (anticholinergics, antidepressants, sedatives, neuroleptics) and nonsteroidal anti-inflammatory drugs.

**Figure 1:**
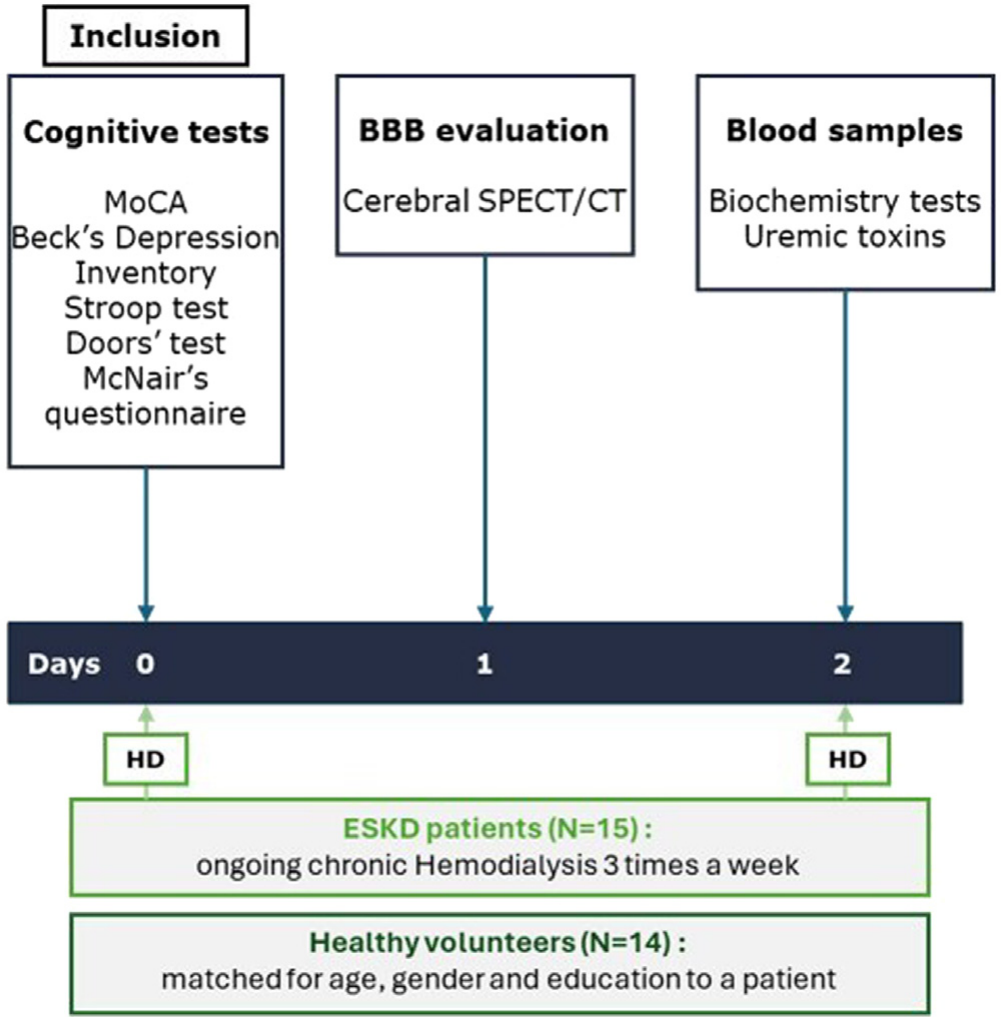
Study design. BBB, blood-brain barrier; ESKD, end-stage kidney disease; HD, hemodialysis session; MoCA, Montreal Cognitive Assessment; SPECT/CT, single-photo emission computed tomography coupled with computed tomography scanner.

The design of the study is provided in Figure 1. Neurocognitive evaluation was performed in all participants at inclusion (D0), during the hemodialysis session in patients with ESKD. Brain SPECT/CT was performed the day after (D1), and blood tests at D2, before the hemodialysis session in patients with ESKD.

### Blood Tests

Classical biochemicals tests, uremic toxins, protein S-100, and NSE were measured in serum. The serum levels of IS, indole acetic acid, and paracresyl sulphate were measured using high performance liquid chromatography using Shimadzu LC2 device.^21^ AhR-AP was quantified in serum by reporter-luciferase gene bioluminescence gene in human HEPG2 cell culture and expressed as a percentage of activation of FICZ (positive control) as previously reported.^22^

### Isotopic Brain Imaging

BBB permeability was assessed using SPECT-CT after injection of an average dose of 750 MBq of (^99m^Tc)- DTPA (Technescan, Curium, France). Brain SPECT/CT was performed 90 minutes after the injection. BBB permeability was quantified by calculating the percentage of total brain uptake of (^99m^Tc)-DTPA (in a whole-brain volume region-of-interest) on injected activity (% IA) by a senior nuclear physician blinded from the kidney function status, using Xeleris workstation (General Electric Healthcare, Waukesha, Wisconsin, USA) and a calibrated factor relating counts per seconds (CPS) to MBq.

### Neurocognitive Evaluation

A battery of 5 neurocognitive tests was performed in all participants, during the beginning of the midweek dialysis session for patients with ESKD, after the first 30 minutes of the session. Global cognitive impairment was assessed by the MoCA,^23^ assessing visuospatial and executive functions, denomination, memory, attention, language, abstraction, and orientation. MoCA score <27 was considered as cognitive impairment. Visual memory was evaluated by the Doors’ test and expressed as the total score obtained (part A + part B).^24^ Attention and interference were evaluated by the Stroop test and expressed as interference T-score.^25^ The existence of depressive patterns was assessed by Beck’s depression inventory, patients with Beck’s depression inventory score >3 was considered to have depression.^26^ The existence of cognitive reliefs in daily living was assessed by the McNair self-administered questionnaire on 49 items.^27^ Overall completion time was about 45 minutes.

#### Statistical Analysis

Qualitative variables are presented in the form of numbers and proportions (percentages), whereas quantitative variables are presented using position and dispersion indicators in the form of mean ± SD, or median and quartiles. A comparative analysis was performed between the 2 groups (patients with ESKD vs. healthy control subjects) using a paired comparison test for qualitative data (McNemar’s test for binary variables, McNemar-Bowker test for nonbinary variables), or a paired comparison test for quantitative data (paired *t* test if valid, Wilcoxon nonparametric test otherwise). The ratio of intracerebral (^99m^Tc)-DTPA activity to total injected activity, the primary end point and marker of BBB permeability, was described and compared between the 2 groups using a matched quantitative data comparison test (matched *t* test). A correlation between continuous variables was estimated using Spearman’s correlation coefficient with its 95% confidence interval. All tests were 2-sided. *P* value below 0.05 was considered significant. Statistical analyses were performed using IBM SPSS Statistics for Windows, Version 24.0.

## Results

Thirty participants were included between December 2020 and January 2023: 15 patients with ESKD and 15 matched healthy volunteers; 1 control was secondarily excluded (wrongly included, taking antidepressive drugs). Therefore, 28 matched participants were included in the paired comparative analyses, and the 15 patients with ESKD were included in the descriptive and correlation analyses. The median age was 50 years. Detailed baseline characteristics of the participants are presented in Table 1. Hemodialysis settings of patients with ESKD are reported in Supplementary Table S1.

**Table 1:** Initial characteristics of participants

Characteristics	Patients with ESKD (*n* = 14)	Matched controls (*n* = 14)	*P* value
Demographic data			
Age, yr	50.6 ± 13.6	50.1 ± 13.9	0.558
Men, *n* (%)	8 (57.1)	8 (57.1)	0.791
Level of education			0.368
Junior high school or less	3 (21.4)	3 (21.4)	
High school or equivalent	5 (35.7)	5 (35.7)	
Bachelor’s degree or more	6 (42.9)	6 (42.9)	
Currently working	1 (7.1)	12 (85.7)	0.001
Past pregnancy (% in women)	4 (66.0)	4 (66.0)	>0.999
Body Mass Index, kg/m^2^	24.4 ± 6.0	25.6 ± 6.0	0.660
Active smoking	2 (14.3)	4 (28.6)	0.687
Systolic blood pressure (mm Hg)	143.2 ± 22.6	127.4 ± 14.2	0.026
Diastolic blood pressure (mm Hg)	77.4 ± 16.0	77.9 ± 7.7	0.889
Hypertension	10 (71.4)	3 (21.4)	0.039
Diabetes	4 (28.6)	0 (0.0)	0.125
Dyslipidemia	2 (14.3)	1 (7.1)	>0.999
Coronary artery disease	4 (28.6)	0 (0.0)	0.125
Congestive heart failure	3 (21.4)	0 (0.0)	0.250
Peripheral artery disease	1 (7.1)	0 (0.0)	>0.999
Ancient neoplasia	1 (7.1)	0 (0.0)	>0.999
Preeclampsia (% in women)	0 (0.0)	0 (0.0)	>0.999
Serum blood tests			
Sodium, mmol/l	138.3 ± 2.8	139.3 ± 1.3	0.161
Potassium, mmol/l	4.9 ± 0.7	3.9 ± 0.2	0.003
Chloride, mmol/l	99.4 ± 4.1	104.8 ± 2.3	0.004
Bicarbonate, mmol/l	19.7 ± 2.5	24.5 ± 1.9	0.001
Albumin, g/l	37.9 ± 4.2	44.8 ± 3.7	<0.001
Calcium (total), mmol/l	2.23 ± 0.13	2.36 ± 0.07	0.005
Phosphate, mmol/l	2.13 ± 1.14	1.00 ± 0.16	0.001
Uric acid, μmol/l	440.2 ± 57.6	301.8 ± 75.7	0.004
Urea, mmol/l	24.3 ± 7.6	5.0 ± 1.6	0.001
Creatinine, μmol/l	946.0 ± 349.5	76.6 ± 12.0	<0.001
Uremic toxins			
Indoxyl sulphate (mmol/l)	86.1 ± 48.4	3.2 ± 1.7	0.001
Indole acetic acid (mmol/l)	2.91 ± 2.34	0.94 ± 0.66	0.003
Paracresyl sulphate (mmol/l)	71.8 ± 58.5	9.0 ± 12.0	0.001
AhR activating potential (% FICZ)	37.7 ± 18.4	24.7 ± 10.4	0.002

AhR, Aryl hydrocarbon receptor; ESKD, end-stage kidney disease.

Qualitative variables are expressed as *n* (%).

Quantitative variables are expressed as mean ± SD.

### Blood-Brain Barrier Permeability is Impaired During CKD

**Figure 2:**
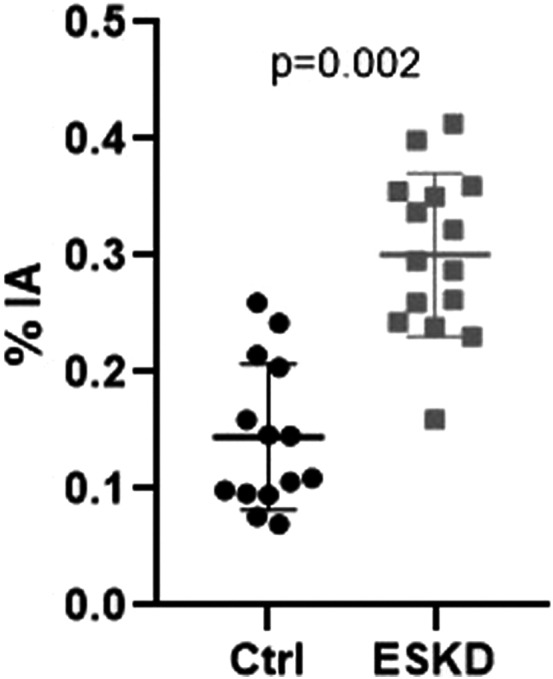
Blood-brain barrier permeability by brain (^99m^Tc)-DTPA SPECT/CT imaging in patients and controls. Ctrl, controls (healthy volunteers); ESKD, end-stage kidney disease; % IA, percentage of injected activity.

**Figure 3:**
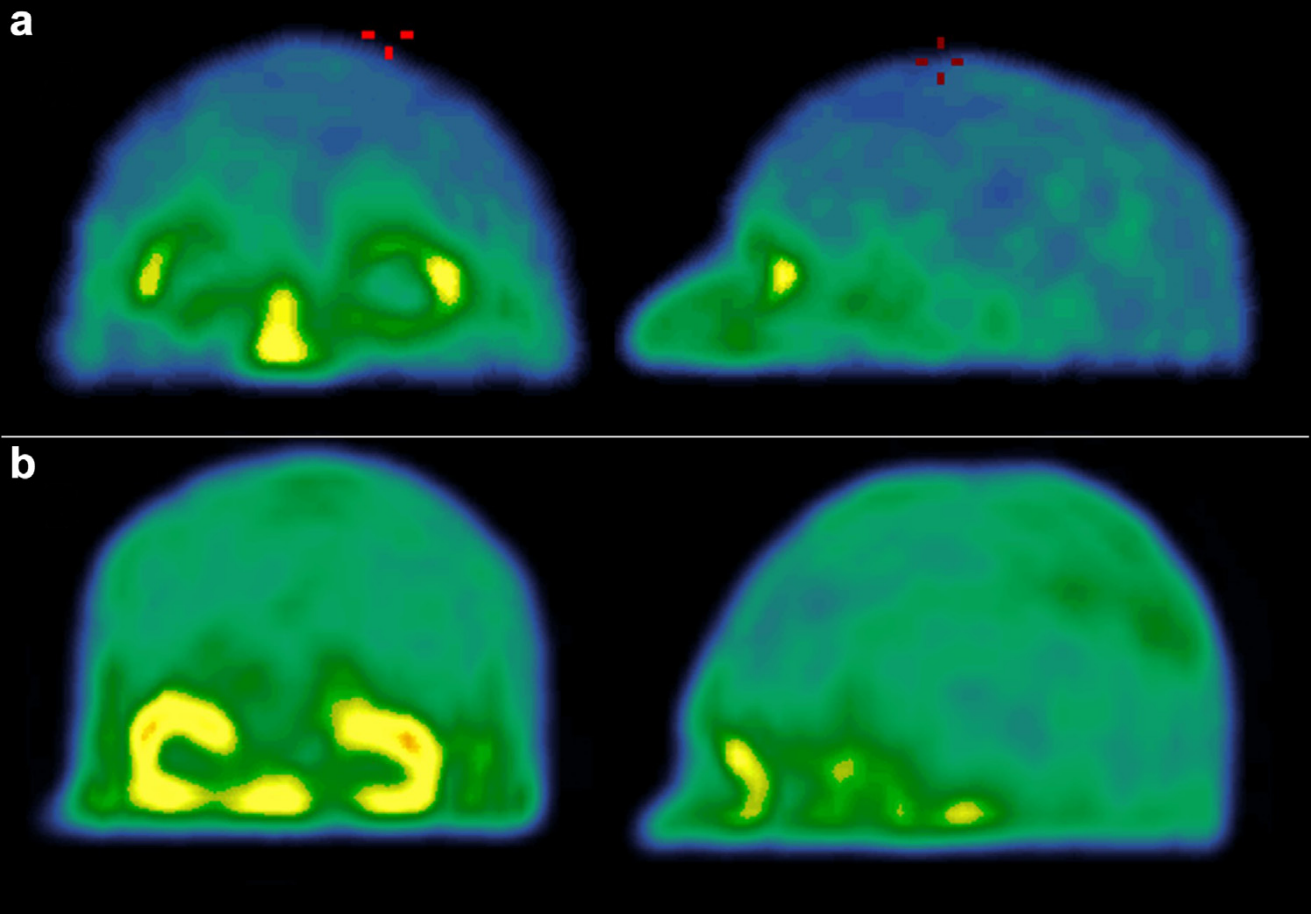
Front and profile 3D maximal intensity projection (MIP) of (^99m^Tc)-DTPA SPECT/CT of a healthy volunteer (A; % IA = 0.0697) and a patient with end-stage kidney disease (B; % IA = 0.4136).

Brain uptake of (^99m^Tc)-DTPA was higher in patients with ESKD: 0.29 ± 0.07 versus 0.14 ± 0.06 % IA, *P* = 0.002, meaning increased BBB permeability compared to matched controls (Figure 2 and 3, Table 2). These results were similar in male participants only: 0.32 ± 0.08 versus 0.15 ± 0.06 % IA, *P* = 0.039, and in female participants: 0.27 ± 0.03 versus 0.14 ± 0.07 % IA, *P* = 0.031 (Supplementary Figure S1). No correlation was found between BBB permeability and dialysis vintage (*r* = −0.36 95% confidence interval (−0.207 to 0.742), *P* = 0.192).

**Table 2:** Blood-brain barrier permeability and cognitive tests in patients and controls

Characteristics	Patients with ESKD (*n* = 14)	Matched controls (*n* = 14)	*P* value
Blood-brain barrier permeability quantification			
(^99m^Tc)-DTPA brain uptake (% IA)	0.29 ± 0.07	0.14 ± 0.06	0.002
Cognitive tests			
MoCA, total score (/30)	21.9 ± 5.1	27.3 ± 2.8	0.007
Cognitive impairment (MoCA <27)	11 (78.6)	3 (21.4)	0.008
MoCA visuospatial / executive	3.4 ± 1.6	4.1 ± 1.2	0.065
MoCA denomination	2.7 ± 0.6	2.9 ± 0.3	0.180
MoCA memory	2.5 ± 1.7	3.9 ± 1.3	0.045
MoCA attention	4.1 ± 1.8	5.8 ± 0.6	0.014
MoCA language	1.7 ± 1.0	2.8 ± 0.4	0.004
MoCA abstraction	1.3 ± 0.8	1.6 ± 0.5	0.160
MoCA orientation	5.9 ± 0.3	5.9 ± 0.3	>0.999
Cognitive reliefs (McNair total score)	61.1 ± 38.2	40.1 ± 17.9	0.060
BDI score	8.7 ± 9.1	2.7 ± 3.4	0.021
Depression (BDI >3)	8 (57.1)	4 (28.6)	0.125
Doors’ test (T-score)	12.5 ± 3.5	16.5 ± 3.7	0.005
Stroop test (T-score)	52.3 ± 9.6	51.9 ± 7.3	0.937
Serum blood-brain barrier tests			
NSE (ng/ml)	19.2 ± 4.1	16.4 ± 6.6	0.132
S-100 (μg/l)	0.08 ± 0.06	0.08 ± 0.04	0.944

BDI, Beck depression inventory; ESKD, end-stage kidney disease; MoCA, Montreal Cognitive Assessment; NSE, Neuron specific enolase.

Qualitative variables are expressed as *n* (%).

Quantitative variables are expressed in mean ± SD.

### Uremic Toxin Accumulation

Patients with ESKD displayed higher serum levels of uremic toxins, IS (86.1 ± 48.4 vs. 3.2 ± 1.7 μmol/l, *P* = 0.001), indole acetic acid (2.91 ± 2.34 vs. 0.94 ± 0.66 μmol/l, *P* = 0.003), and paracresyl sulphate (71.8 ± 58.5 vs. 9.0±12.0 μmol/l, *P* = 0.001) than matched controls. AhR activating potential (AhR-AP) was higher in the serum of patients with ESKD (37.7 ± 18.4 vs. 24.7 ± 10.4 %, *P* = 0.002) (Table 1). No correlation was found between Brain uptake of (^99m^Tc)-DTPA and serum uremic toxin levels in patients with ESKD (Supplementary Table S2).

### Cognitive Impairment

Patients with ESKD had lower MoCA scores: 21.9 ± 5.1 versus 27.3 ± 2.8, *P* = 0.007, with significant lower scores in memory, attention, and language compared to controls, and 78.6% of there were considered to have cognitive impairment according to MoCA test (vs. 21.4% of controls, *P* = 0.008). They displayed slightly more cognitive reliefs (McNair total score: 61.1 ± 38.2 vs. 40.1 ± 17.9, *P* = 0.060), and higher scores at Beck’s Depression Inventory: (8.7 ± 9.1 vs. 2.7 ± 3.4, *P* = 0.021). Interference T-score with Stroop test was not different between the 2 groups (Table 2). No correlation was found between brain uptake of (^99m^Tc)-DTPA any of the scores in cognitive tests in patients with ESKD (Supplementary Table S2). However, MoCA score was correlated with brain uptake of ^99m^Tc-DTPA in the overall population (*r* = −0.60 95% confidence interval (−0.772 to −0.339), *P* = 0.001) (Supplementary Figure S2). NSE and protein S-100 levels were not different between the 2 groups.

## Discussion

Our proof-of-concept study highlights the increase of BBB permeability in patients with ESKD confirming our previous results in rodent models of CKD.^13^ Interestingly, we found the same level of increased DTPA uptake in our patients as in the rat and mouse models we had used previously.

(^99m^Tc)-DTPA brain SPECT/CT appears as a reliable and sensible imaging technique for evaluating the BBB permeability in humans. This imaging technique could be more largely used to evaluate chronic BBB disruption, notably in the context of chronic systemic diseases with neurocognitive impairment. In a cross-sectional study including young patients with CKD and healthy controls, the differences in brain volumes were subtle and no association was found between brain volumes in structural magnetic resonance imaging and neurocognitive performance.^28^ Brain injury serum biomarkers S-100 protein and neuron-specific enolase appear poorly discriminative to assess BBB dysfunction in patients with CKD in our study. This may reflect a subtle chronic BBB disruption rather than an abrupt one as in stroke, because ^99m^Tc-DTPA is very sensitive to detect BBB disruption, but also possibly a lack of statistical power. Winter *et al.*^29^ found no correlation between S-100 levels and BBB disruption quantified by ^99m^Tc-DTPA SPECT/CT and dynamic contrast-enhanced magnetic resonance imaging in patients with traumatic brain injury.^29^

In our overall population, we highlighted a significant inverse correlation between brain uptake of (^99m^Tc)-DTPA and MoCA scores, suggesting a link between BBB permeability and cognitive impairment. In CKD, the association of BBB permeability with cognitive impairment needs to be confirmed in larger dedicated studies. BBB permeability should also be evaluated in earlier stages of CKD, to identify whether this mechanism appears early in CKD or mainly in Patients with ESKD.

Kidney transplantation has been shown to partially improve the cognitive function of patients with CKD ^30^ and their brain networks,^31^ particularly episodic and verbal memory;^32^ however, its impact on BBB is also unknown. However, Hernandez *et al.*,^33^ reported that serum levels of BBB biomarkers such as NSE, brain-derived neurotrophic factor or neurofilament light chain will decrease after kidney transplantation.^33^ Longitudinal studies evaluating the evolution of the BBB permeability and its reversibility after transplantation are needed.

BBB is a complex barrier with high selectivity formed by nonfenestrated cerebral endothelium displaying numerous tight junctions, basal membrane, pericytes, and astrocyte feet.^15^ The exact mechanism underlying BBB disruption in CKD remains unclear, and is probably multifactorial,^8^ involving patients’ medical conditions, notably hypertension, modification of cerebral blood flow during hemodialysis, and accumulation of uremic toxins. The absence of association between BBB permeability and dialysis vintage may suggest a process already present in patients with CKD, and related to the uremic state, rather than specifically related to dialysis. We found that AhR activation by IS accumulation in CKD models in rodents is linked to BBB disruption and impaired cognitive performance,^13^ suggesting a AhR-induced cerebral endothelial dysfunction. However, in the present study, although we found higher uremic toxin levels and AhR activation in patients with ESKD compared to healthy volunteers, we could not highlight any significant correlation between AhR activation or uremic toxins activation and the intensity of BBB permeability in our small population of patients with ESKD.

This chronic BBB disruption in patients with ESKD may have numerous adverse effects and could partially explain the higher prevalence of cognitive impairment, white-matter lesions,^34^ asymptomatic cerebral microbleeds,^35^ increased severity of stroke,^34^^,^^36^ and higher drug neurotoxicity^37^^,^^38^ observed in this population. Its specific impact on brain cell populations and neuroinflammation is also unknown in humans, although experimental work suggests a link between IS accumulation and neuroinflammation *in vitro* and *in vivo*.^12^^,^^39^

Recently, the study of Gupta *et al.*^40^ found an increased BBB permeability in patients with ESKD, with a similar methodology of BBB permeability quantification by cerebral (^99m^Tc)-DTPA, SPECT/CT.^40^ We observed cerebral uptake values in our patients and controls close to theirs. This suggests the reliability of this method to evaluate the BBB permeability in patients with CKD. Our study confirms these findings in a larger cohort and allows us to investigate further on neurocognitive impairment in patients with ESKD. We performed a large battery of cognitive tests allowing us to explore various aspects of cognition and thymic disorders.

We highlighted a major and alarming prevalence of previously undiagnosed cognitive impairment in a relatively young population of patients with ESKD, with 78.6% of patients displaying at least mild cognitive impairment according to the MoCA test. We found that among cognitive functions, memory, attention, and language were the most impacted functions of cognition in patients with ESKD, as highlighted by the MoCA and Doors’ tests, while orientation and abstraction were relatively preserved. This is consistent with a recent epidemiological study which found that lower estimated glomerular filtration rate was associated with impaired praxis, attention, and language features in 3003 patients with CKD.^41^ A previous study found that IS levels, but not paracresyl sulphate levels, were associated with lower scores in MOCA in patients with ESKD.^42^ We also found a high prevalence of depressive features in patients with ESKD (57.1%), previously undiagnosed for depression. A previous large meta-analysis also highlighted a high prevalence of depression between 22.8% and 39.3% in patients with ESKD, depending on the tools used for diagnosis.^43^

Our study has several limitations. It is a pilot study with a limited number of patients. Although we highlighted a negative correlation between MoCA score and BBB permeability in our overall population, suggesting BBB could be one of the mechanisms of cognitive impairment, we did not find any significant correlation between scores in cognitive tests and BBB permeability in patients with ESKD, not allowing us to conclude a causal relationship between BBB disruption and cognitive impairment in patients with ESKD. This is certainly due to limited statistical power. We chose to perform cognitive tests during hemodialysis sessions to minimize disturbance to patients. This may possibly have an impact on their results on cognitive tests, because of a decrease in cerebral blood-flow during hemodialysis, especially during the first hour of the session.^44^ Finally, comorbidities such as coronary artery disease or diabetes^18^ of patients with ESKD may also have an impact on BBB permeability and cognitive troubles, although these comorbidities insufficiently explain the major prevalence of cognitive troubles in patients with CKD.^7^ Patients with ESKD also displayed hypertension more frequently, which can contribute to brain alterations,^17^ although patients with nonequilibrated hypertension were excluded.

## Conclusion

This proof-of-concept study confirms that patients with ESKD display an increased BBB permeability compared to matched healthy volunteers, as well as highly prevalent cognitive impairment. BBB disruption may be an important mechanism of cognitive impairment in CKD. Association of BBB dysfunction with uremic toxins levels and cognitive impairment needs to be assessed in larger cohorts.

## Disclosure

All the authors declared no competing interests.

## Supplementary Material

Supplementary File (PDF) **Figure S1.** Sex-disaggregated analysis of blood-brain barrier permeability by brain (^99m^Tc)-DTPA SPECT/CT imaging in male and female patients and controls. **Figure S2.** Correlation between (^99m^Tc)-DTPA brain uptake and MoCA score in the overall population with 95% confidence interval. **Table S1.** Hemodialysis characteristics and settings of patients with end-stage kidney disease. **Table S2.** Correlation between (^99m^Tc)-DTPA brain uptake and uremic toxins and scores in cognitive tests in patients with chronic kidney disease. **STROBE Statement.**

## Footnotes

Supplementary File (PDF)
**Figure S1.** Sex-disaggregated analysis of blood-brain barrier permeability by brain (^99m^Tc)-DTPA SPECT/CT imaging in male and female patients and controls. **Figure S2.** Correlation between (^99m^Tc)-DTPA brain uptake and MoCA score in the overall population with 95% confidence interval. **Table S1.** Hemodialysis characteristics and settings of patients with end-stage kidney disease. **Table S2.** Correlation between (^99m^Tc)-DTPA brain uptake and uremic toxins and scores in cognitive tests in patients with chronic kidney disease.
**STROBE Statement.**
